# North Texas Medical Association, the Coming Meeting

**Published:** 1890-11

**Authors:** V. A. Howeth, Geo. R. Clayton

**Affiliations:** President, Gainesville, Texas; Sec’y.; Sherman, Texas


### NORTH TEXAS MEDICAL ASSOCIATION.

GAINESVILLE, TEXAS, November 15, 1890.

DEAR DOCTOR: The next session of the North Texas Medical Association will be held in the City of Sherman, beginning on Tuesday, December 9, 1890, promptly at 3 p. m., and continue for three days.

All those contributing papers or reports of cases, will please inform the Secretary immediately upon their arrival, giving the name and title of papers. If any who cannot attend have papers, please send them to the Secretary by mail or otherwise.

Contributions of exceptional interest from prominent medical gentlemen outside of the State have been promised.

This is a wide-awake age of pronounced energy, investigation and advancement in all things—the profession of medicine has not been slothful or inactive, but keeps well apace, if not in the van. Our Association is the vital center of organized regular medicine in North Texas. It is a crystal fountain whose sparkling waters all may quaff and feel and enjoy its exhilarating and invigorating powers. The older members have learned to love the Association with a love that widens and deepens and intensifies with time, and they especially wish and urge the young to come forward in the work of perfecting, building up and perpetuating an association noted for its uniform harmony, practical teachings and valuable work and labor.

Let us take inspiration and enthusiasm worthy our seniors and push forward the work so nobly begun.

Every worthy, regular physician is eligible to membership and the time has come in Texas when every physician must work and belong to some live working society, or be considered as professionally moribund, or classed among the non-progressive who fail in their duty to themselves, their profession and the public as well.

Our Association is a veritable school where the old teach the young, and the young the old. Its future is as promising and bright as its past has been as successful and brilliant. Our meeting and commingling on these occasions brings the profession closer together and enables its members to form extensive and pleasant acquaintanceship, affording the choicest social enjoyments and is a means of awakening and engendering the most enjoyable and enduring friendshps.

You observe we have a good programme and will have an interesting session. You are are cordially invited to attend and take part in the proceedings.

V. A. HOWETH, M. D., President,

Gainesville, Texas.

GEO. R. CLAYTON, M. D., Sec'y.

Sherman, Texas.

SECTION ON PRACTICE OF MEDICINE.

Rational Medicine—Wm. R. Howard, M. D., Fort Worth.

Medication and Diet in Some of the Diseases of Children—R. D. Potts, M. D., Bonham.

Rheumatic Fever, a Survey of its Pathology and Complications—W. R. Mathers, M. D., Collin county.

SECTION ON OBSTETRICS AND GYNECOLOGY.

Chloroform During Labor—S. C. Relyear, M. D., Ladonia.

The Management of the Lying-in Period—G. W. Bedford, M. D., Paris.

Antiseptic Midwifery—O. C. Buster, M. D. Lewisville.

SECTION ON SURGERY.

Surgical Shock—J. B. Stinson, M. D., Sherman.

The Diagnosis and Treatment of Surgical Diseases of the Thoracic Cavity—Bacon Saunders, M. D., Bonham.

Antiseptics—Henry R. Smith, M. D., Woodland.

VOLUNTEER PAPERS.

The Continued Fevers of North Texas, Presenting no Well Defined Specific Characteristic. What are They? Their Taeatment—V. A. Howeth, M. D., Gainesville, President of the Association.

Chloroform in Puerperal Eclampsia—Alonzo Sims, M. D., Weatherford.

Nasal Catarrh—J. B. Hill, M. D., McKinney.

The Surgery of Homer's Iliad—Jno. O. Scott, M. D., Sherman.

Two cases of Metastatic Abscess—J. B. Stinson, M. D., Sherman.

